# Nanoparticle-Delivered HIV Peptides to Dendritic Cells a Promising Approach to Generate a Therapeutic Vaccine

**DOI:** 10.3390/pharmaceutics12070656

**Published:** 2020-07-11

**Authors:** Alba Martín-Moreno, José L. Jiménez Blanco, Jamie Mosher, Douglas R. Swanson, José M. García Fernández, Ajit Sharma, Valentín Ceña, María Angeles Muñoz-Fernández

**Affiliations:** 1 Section of Immunology, ImmunoBiology Molecular Laboratory, Spanish HIV HGM BioBank, Hospital General Universitario Gregorio Marañón, 28007 Madrid, Spain; albamartinm35@gmail.com; 2 Department of. Química Orgánica, Facultad de Química, Universidad de Sevilla, 41012 Sevilla, Spain; jljb@us.es; 3 Department of Chemistry & Biochemistry, Central Michigan University, Mount Pleasant, MI 48859, USA; mosserj@cmich.edu (J.M.); swansond@cmich.edu (D.R.S.); sharm1a@cmich.edu (A.S.); 4 Institute for Chemical Research, CSIC–University of Sevilla, 41092 Sevilla, Spain; jogarcia@iiq.csic.es; 5 CIBERNED, Instituto de Salud Carlos III, 28031 Madrid, Spain; valentin.cena@gmail.com; 6 Unidad Asociada Neurodeath, Facultad de Medicina, Universidad de Castilla-La Mancha, 02006 Albacete, Spain; 7 Networking Research Center on Bioengineering, Biomaterials and Nanomedicine (CIBER-BBN), Instituto de Salud Carlos III, 28034 Madrid, Spain

**Keywords:** HIV-1, DCs, polycationic nanoparticles, delivery, fluorescence peptides, cytokines, vaccine

## Abstract

Finding a functional cure for HIV-1 infection will markedly decrease the social and economic burden of this disease. In this work, we have taken advantage of the antigen presenting cell role of human dendritic cells (DCs) to try to induce an immune response to HIV-derived peptide delivered to DCs using two different polycationic nanoparticles: a G4 PAMAM dendrimer modified to a 70/30 ratio of hydroxyl groups/amines and a cyclodextrin derivative. We have studied peptide delivery using a fluorescence peptide and have studied the immune response generation by cytokine determination and flow cytometry. We have found a robust delivery of the antigenic peptide to DCs and activated dendritic cell-mediated peripheral blood mononuclear cells (PBMCs) proliferation using the mixed lymphocyte reaction. However, no expression of markers indicating activation of either B or T lymphocytes was observed. Moreover, the release of the pro-inflammatory cytokine TNF-α or IL-2 was only observed when DCs treated with either the dendrimer or the dendriplex containing the peptide. Antigenic peptide delivery to DCs is a promising approach to generate a vaccine against HIV-1 infection. However, more studies, including the simultaneous delivery of several antigenic peptides from different viral proteins, can markedly improve the immune response.

## 1. Introduction

Since the development and commercialization of anti-retroviral therapy (ART), HIV-1 infection is no longer a death sentence and has become a chronic infection for those patients with access to treatment [1,2,3]. According to the Joint United Nations Programme on HIV and AIDS (UNAIDS) data, 23.3 million people (out of the 38 million people infected with HIV-1) were accessing ART [4]. Access to available treatment and adherence to it are key factors that determine the fate of the patients, as viral load and lymphocyte death rapidly increase upon treatment interruption. Economic issues may explain the scarcity of treatment available in a significant number of countries, and the wide range of social stigmas and psychological side-effects might be responsible for the lack of adherence to ART. The average annual cost of ART per person was estimated to be $19,912 in 2006 and $23,000 in 2010, according to the US Center for Disease Control and Prevention (CDC), which results on an estimate average of about $380,000 for a lifetime [5]. For these reasons, finding a more accessible and long-lasting prevention/therapeutic approach against HIV-1 infection is one of the biggest current challenges of the biomedical community.

Viral latency, high mutability, and variability highly hinder the finding of a cure or an efficient prophylactic vaccine [6,7], making the possibility of either option controversial among researchers [8,9]. In the absence of a complete cure for HIV-1 infection, which remains elusive, a functional cure seems a promising option to decrease expenses, reach a higher percentage of the HIV-infected population and most importantly, improve the quality of life of HIV-1 infected people. Functional cure means that HIV-1 is still present in the host organism and remains latent in the genome of many cells, but the host immune system keeps the infection under control in the absence of treatment. This requires a specific and efficient immune response.

Therapeutic vaccines, even being not fully effective might contribute to HIV therapy since its use early post-infection might limits HIV reservoir size and contribute, in addition to ART, to viral eradication. During the last 30 years, a significant number of vaccine candidates have been tested with disappointing results [10]. A new strategy consisted in inserting HIV genes into recombinant viral vectors and shuttle these genes into the Class I antigen-presenting pathway [11]. This has led to the exploration of novel ways of antigen administering including adenovirus 5-mediated delivery of DNA without positive results [12]. Another approach for developing HIV vaccines has led to the use of nanoparticles as delivery agents for HIV antigenic peptides to antigen presenting cells (APCs) to elicit a therapeutic response [13]. We explored this approach using a cationic phosphorous dendrimer which failed to induce an efficient antigenic response [13]. Since gag-derived peptides (including p24-derived peptides) have potential antigenic activity [14], we decided to explore whether other NPs with different chemical structure were able to deliver the peptide in such a way that it would produce a significant antigenic response. This was the rationale to perform the present experiments since we think that NPs can be very helpful tools to potentiate the antigenic presentation and so contribute to elicit a potent immune response.

Taking advantage of the APC role of DCs [15,16], we aimed to design a therapeutic vaccine against HIV-1. Our objective was to extract human DCs from buffy coats, and modify them to become efficient HIV-1-specific APCs with the ability to generate a strong T cell response when introduced back to the patient. For this purpose, we used cationic nanocompounds, G4-70/30 dendrimer and the β-cyclodextrin derivative AMC6, to introduce HIV-1 peptides to the DCs and study their maturation and T cell-activation.

## 2. Materials and Methods

### 2.1. Nanocompounds

We have designed and synthesized a fourth generation PAMAM dendrimer (G4-70/30) (Supplementary Figure S1), with the peripheral residues modified to a 70/30 ratio of hydroxyl groups/amines, which decreases its toxicity in spite of the size and total amount of surface residues, while still presenting enough positive charges to bind RNA strands or peptides. The same dendrimer, but with a 90/10 hydroxyl groups/amines ratio had already been shown to be non-toxic in mice and able to cross the blood-brain barrier (BBB) [17,18]. Mannose residues (α-d-mannopyranosylphenyl isothiocyanate, Sigma-Aldrich, St. Louis, MO, USA) were added to the G4-70/30 dendrimer to target DCs (the amount of α-d-mannopyranosylphenyl isothiocyanate added was between 10% and 20% compared to the amount of amine groups present in the dendrimer). G4-70/30 dendrimer was labeled with Cy5.5 (Lumiprobe, Hallandale Beach, FL, USA), in order to allow its visualization (G4-7/30-Cy5.5).

AMC6 is a monodispersed macromolecular nanocompound consisting of a basket-shaped polyanionic amphiphilic β-cyclodextrin core bearing seven tetraethyleneimine branches at the primary (narrower) rim, ending with 28 protonable nitrogens, and 14 hexanonyl chains at the opposite secondary face (Supplementary Figure S2). The synthesis of AMC6 involves a multiple copper(I)-catalyzed azide-alkyne cycloaddition (CuAAC) ‘click’-type reaction, resulting in the formation of triazole linkers [19]. AMC6 is able to bind negatively charged compounds such as siRNA forming complexes due to electrostatic interactions between siRNAs and nanocompounds [20].

### 2.2. Primary Cell Cultures, Purification and Differentiation

#### 2.2.1. Human Peripheral Blood Mononuclear Cells

PBMCs were isolated from buffy coats obtained from anonymous healthy blood donors donated by the Transfusion Center of Madrid following national guidelines. PBMCs were isolated by a standard Ficoll-Hypaque density gradient (Rafer, Madrid, Spain) and cultured following the procedures of Spanish HIV HGM BioBank [21,22,23]. PBMCs were cultured in RPMI-1640 medium (Biochrom AG, Berlin, Germany) with 10% fetal bovine serum (FBS; Gibco, London, UK), 1% l-glutamine (Lonza, Walkersville, MD, USA) and antibiotic cocktail (125 mg/mL ampicillin,125 mg/mL cloxacillin and 40 mg/mL gentamicin; all from Normon, Madrid, Spain). Interleukin-2 (rhIL-2; Bachem AG, Bubendorf, Switzerland), was added at 60 U/mL to ensure T cell survival, and phytohemagglutinin (PHA; Remel, Santa Fe, CA, USA) at 1 µg/mL was used as T cell activation control.

#### 2.2.2. Monocytes and Monocyte-Derived Dendritic Cells

Monocytes were purified using immune-magnetic anti-CD14 microbeads (Miltenyi, Bergisch Gladbach, Germany). They were seeded at 10^6^ cells/mL in RPMI-1640 medium supplemented with 10% FBS, 1% l-glutamine, 50 μM β-2-mercaptoethanol (Sigma-Aldrich, St. Louis, MO, USA), 20 ng/mL recombinant human interleukin 4 (rhIL-4; Immunotools, Friesoythe Germany) and 50 ng/mL recombinant human granulocyte macrophage colony stimulating factor (rh GM-CSF; Immunotools, Friesoythe, Germany), and were maintained in culture for 5 consecutive days. Culture medium was renewed the third day. iDCs were cultured for 48 h in the presence of CD40L (500 ng/mL) (hCD154, CD40 Ligand; Invitrogen, Carlsbad, CA, USA) and TNF-α (50 ng/mL) (rh TNF-alpha, Immunotools, Friesoythe Germany). Incubation with lipopolysaccharide (LPS; Sigma-Aldrich, St. Louis, MO, USA) at 20 ng/mL was used as control of DC maturation.

### 2.3. HIV-1 Derived Peptide

HIV-derived peptide was synthesized by Eurogentec (Sereing, Belgium). The peptide was chosen from HIV-HXB2 location in Gag-p24 (71–80, NH2-DTINEEAAEW-COOH) which is an HLA-A25 epitope from a conserved region of the p24 gag protein [24]. Although this peptide is only part of the protein, the name of complete protein was used to refer to it (p24). The p24 peptide has a molecular weight of 1177.17 g/moL, and net charge of –4 at pH 7, and although it contains 4 hydrophobic amino acids, it has good solubility in water [13,25].

### 2.4. Cell Viability Assay

The cytotoxicity of G4-70/30 dendrimer and AMC6 nanoparticle on DCs (7.5 × 104 cells/200 μL) was tested in vitro by MTT assays. MTT assay is a colorimetric assay based on the ability of living cells to reduce 3-(4,5-dimethylthiazol-2-yl)-2,5-diphenyl tetrazolium bromide to form soluble formazan crystals. This assay was carried out according to the manufacturer’s instructions (MTT, Sigma-Aldrich, St. Louis, MO, USA). Briefly, the cells were treated with increasing concentrations of the dendrimers and, after 24, 48, or 72 h of incubation, the supernatant containing the dendrimer was removed and replaced with 200 μL of Opti-MEM^®^ (Gibco, London UK) containing 0.5 mg/mL of MTT substrate. After 2 h of incubation under cell culture conditions, the plate was centrifuged at 2000 rpm for 10 min, and the supernatants were discarded. The formazan crystals were diluted in 200 μL of dimethyl sulfoxide (DMSO; Sigma-Aldrich, St-Louis, MO, USA). The plate was shaken at 700 rpm and formazan concentrations were determined by spectrophotometry (Synergy 4 Plate Multileaver, Biotek Instrument, Winooski, USA) at a wavelength of 570/690 nm. The spectrophotometer was calibrated at 0 using only Opti-MEM^®^. Absorbance values were interpreted as a measurement of cell viability. DMSO was used as a positive control of cell death. Each experiment was performed by triplicate.

### 2.5. Flow Cytometry

Flow cytometry was performed as previously described [26,27]. Analysis of the expression levels of cell-surface markers was performed by flow cytometry. DCs were blocked for 5 min with PBS containing 10% heat inactivated human AB serum (Sigma-Aldrich, St. Louis, MO, USA), and then stained by adding the antibodies to the same buffer. All other cell types were stained on PBS containing 3% FBS. Cells were labeled with anti-CD1a-PE, anti-CD3-PC7, anti-CD4-FITC, anti-CD14-PC7, anti-CD19-ECD, anti-CD25-PC7, anti-CD69-PC5, anti-CD80-FITC, anti-CD86-PC5.5, anti-CD86-PE, anti-HLA-DR-ECD, anti-HLA-DR-PB, anti-HLA-DR-PE (all from Beckman Coulter Inc., Brea, CA, USA), anti-CD8-Pacific Blue, anti-CD71-APC-Cy7, anti-CD197-APC-Cy7 (CCR7) (all from BioLegend, San Diego, CA, USA), anti-CD83-APC (BD Biosciences, Franklin Lake, NJ, USA), and anti-CD3-PO (Abcore, Ramona, CA, USA).

Levels of surface expression on cells were estimated by flow cytometry (Gallios; Beckman Coulter, Brea, CA, USA) and analyzed using Kaluza software (Beckman Coulter, Brea, CA, USA). Entry of FITC-labeled p24 peptide into the DCs was also estimated by flow cytometry. DCs were collected 2 h, 24 h, and 48 h after complex addition, washed with PBS, and acid washed, in order to eliminate any peptide attached to the cell surface. Fluorescence was measured by flow cytometry.

### 2.6. Complex Formation and Electrophoretic Measurements

Dendrimer-peptide complexes were formed by mixing p24 peptide and dendrimers dissolved in ddH2O at ten times the desired final concentration for cell culture (1 μM p24) and incubated for 24 h at RT. A fixed concentration of 1 µM of peptide was used in all assays. Complex formation was evaluated as migration retardation of FITC-labeled peptide during electrophoresis on a 7% agarose gel at 90V for 45 min using TEA buffer. After electrophoresis, the fluorescence bands in the gel were visualized using a Biorad Molecular Imager Gel Doc XR System (Hercules, CA, USA).

### 2.7. Dendrimer Visualization by Confocal Microscopy

FITC-labeled peptide and Cy5.5-labeled dendrimer were used to monitor the entry of the complex into the DCs. DCs matured in the presence of the complex for 48 h were collected, washed, fixed, permeabilized and stained with Phalloidin and DAPI. The samples in suspension were observed under the microscope and images were acquired with a Zeiss LSM710 confocal microscope using Zen 2011 software (Carl Zeiss Microimaging Inc., Thornwood, NY, USA).

### 2.8. Autologous Mixed Lymphocyte Reaction

PBMCs and CD14^+^ cells were extracted from the same buffy coat. PBMCs were kept at −80 °C during the 7 days needed for DC differentiation and maturation. Dendrimer-peptide complexes were added to the DCs at the end of the maturation period. After 48 h, PBMCs were thawed and plated at 10^6^ cells/mL, DCs were added at a 1:10 ratio respect to PBMCs, and IL-2 (60 U/mL) was added. The monoclonal antibody nivolumab (Bristol-Myers Squibb, NY, USA) was added, when indicated, at 10 ng/mL, 1 µg/mL and 10 µg/mL to help stimulate T cell activation. Supernatants were collected and lymphocyte activation was analyzed at days 3 and 6 after DCs-PBMCs co-culture.

### 2.9. Bright field Microscopy

Bright field microscopy images were taken using a Leica DMI3000 B microscope and a Leica DFC310FX camera (Leica Camera AG, Wetzlar, Germany). Leica Application Suite (LAS) v4.5 program was used to acquire the images.

### 2.10. Cytokine Quantification

Cytokines were measured from 50 µL supernatants of the MLR samples. The cytokine production profile was measured by DiaclonDIAplex Th1/Th2/Inflammation kit (Gen-Probe Diaclone SAS, BesanCon, France) according to the manufacturer’s protocol. The results were analyzed following Diaplex Pro Software instructions.

### 2.11. Autologous Mixed Lymphocyte Response

PBMCs were extracted from buffy coats of healthy donors and kept frozen at −80 °C for 7 days. Monocytes were purified using immune-magnetic anti-CD14 microbead (Miltenyi, Bergisch Gladbach, Germany). CD14^+^ cells were isolated from another fraction of the same buffy coats and differentiated into DCs following the previously described method. Once the DCs were differentiated, treated, and matured, PBMCs from the same donor were thawed. Cells were mixed at a 1:10 and 1:5 DCs-PBMCs ratio and cultured in 12-well plates in RPMI-1640 medium (Biochrom AG, Berlin, Germany) with 10% fetal bovine serum (FBS; Gibco, London, UK), 1% l-glutamine (Lonza, Walkersville, MD, USA) and antibiotic cocktail (125 mg/mL ampicillin,125 mg/mL cloxacillin and 40 mg/mL gentamicin; all from Normon, Madrid, Spain) with 60 U/mL interleukin-2 (rhIL-2; Bachem AG, Bubendorf, Switzerland). Phytohemagglutinin (PHA; Remel, Santa Fe, CA, USA) at 1 µg/mL was used as T cell activation control.

### 2.12. Reagents

The folllowing sources for reagents were used: dimethyl sulfoxide (DMSO, Sigma-Aldrich, St-Louis, MO, USA); PBS (ThermoFisher, Madrid, Spain); Phytohaemagglutinin (PHA; Remel, Santa Fe, CA, USA). The rest of reagents used were obtained from different sources and were of the maximal purity possible.

### 2.13. Statistical Analysis

Nonparametric variance analysis (Kruskal–Wallis) followed by a Dunn test was used to assess statistical differences between groups. *p* < 0.05 was considered statistically significant. Statistical analyses were performed using the software package SPSS 13.0 (SPSS, Chicago, IL, USA).

## 3. Results

### 3.1. In Vitro Toxicity of AMC6 Nanoparticle and G4-70/30 Dendrimer

**Figure 1:**
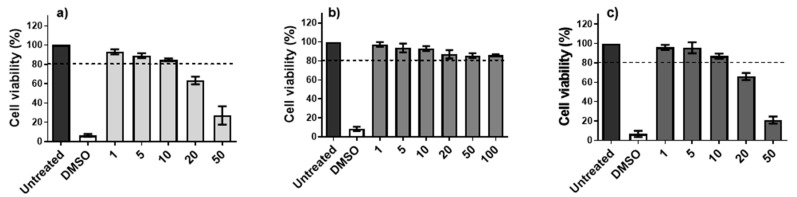
Biocompatibility of AMC6 nanoparticle, G4-70/30 dendrimer, and Cy5.5-labeled G4-70/dendrimer in DCs. The viability of monocyte derived-DCs was evaluated by MTT assay 48 h after exposure to (**a**) AMC6 nanoparticle, (**b**) G4-70/30 dendrimer, and (**c**) G4-70/30-Cy5.5 labeled dendrimer. The limit of toxicity was established at 80% of cell viability (dotted line). 10% DMSO was used as control for cell death. The data represent mean ± standard deviation of 3 different experiments.

The toxicity of AMC6 nanoparticle and G4-70/30 dendrimer on DCs was assessed by MTT assays. Nanoparticle concentrations were considered non-toxic when cell viability after 48 h of incubation decreased below 80%. AMC6 nanoparticle was non-toxic up to 10 µM, while G4-70/30 dendrimer was non-toxic even at 100 µM. Cy5.5-labeled G4-70/30 dendrimer was toxic at 20 µM, allowing for a maximum working concentration of 10 µM (Figure 1).

### 3.2. AMC6 Nanoparticle and G4-70/30 Dendrimer Complex HIV-1 Derived Peptides

**Figure 2:**
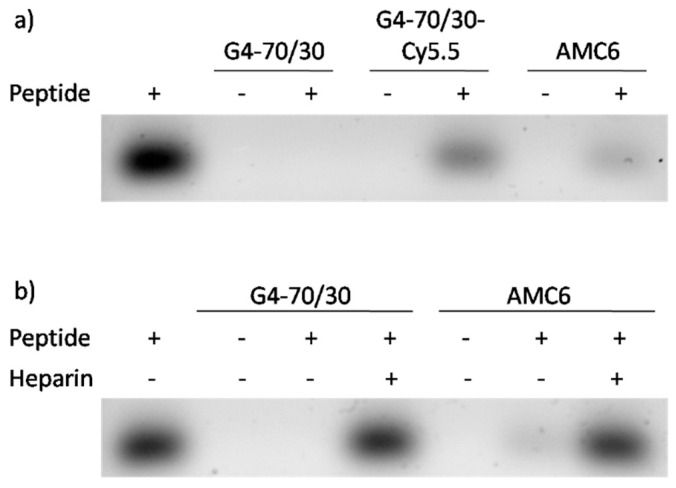
Binding of the fluorescent peptide to the G4-70/30 dendrimer and AMC6 nanoparticle release by heparin. (**a**) Binding of the peptide to G4-70/30 (20 µM), G4-70/30-Cy5.5 (20 µM) and AMC6 (3 µM) increases the size of the complex, decreasing its migration in the agarose gel, and it is visualized as the decrease in the peptide band. Complexes were run on the gel after 24 h of incubation at RT. (**b**) Heparin added to the complex, competes with the peptide for the dendrimer, and causes a release of the peptide.

The positive charges of both nanoparticles allow the formation of complexes with anionic HIV-derived peptides through electrostatic interactions. We used a peptide derived from the p24 protein of HIV-1 (H_2_N-DTINEEAAE W-COOH), which, at pH 7, has a net charge of −4. This peptide had been previously reported to induce an immune response when added to the DCs at 1 µM [13]. The peptide was labeled with fluorescein (FITC) to allow its visualization. After incubation of the peptide (1 µM) with G4-70/30 dendrimer (20 µM); Cy5.5-labeled G4-70/30 dendrimer (20 µM); Cy5.5-labeled G4-70/30 dendrimer (20 C or AMC6 nanoparticle (3 µM) for 24 h and 48 h, the samples were separated by electrophoresis on a 7% agarose gel and observed under UV light. Figure 2a shows that the fluorescent peptide was bound almost completely by the dendrimers although dendrimer decoration with Cy5.5 decreased the dendrimer efficiency to bind the peptide (Figure 2a). Incubation for longer periods of time (48 h) resulted in similar observations, thus suggesting that the complexes were fully formed in 24 h, and remain stable (data not shown).

Once the complex enters the cell, the peptide needs to be released from the dendrimers in order to be processed as an antigen and presented by the DCs. As the complex is formed by electrostatic interactions, the assumption is that negatively charged proteins inside the cell will disrupt the dendrimer-peptide interaction and facilitate its release. Due to its high negative surface charge density, heparin acts as a competitor for the binding of the dendrimer and mimics the effect of negatively charged physiological compounds on releasing the peptide from the dendrimers. Incubation of the complexes with heparin (0.2 USP/μL; 5 min) resulted in a complete release of the peptide from all the nanocompounds (Figure 2b).

### 3.3. Dendrimers are Effective Carriers for Peptides into the Dendritic Cells

**Figure 3:**
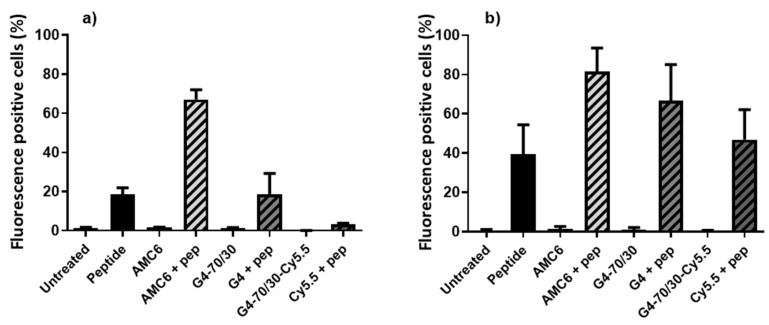
Entry of FITC labeled peptide into the DCs observed by flow cytometry. The nanoparticles, peptide and complexes were added to the DCs, and green fluorescence was quantified at (**a**) 2 h and (**b**) 24 h post-treatment. The percentage of FITC positive cells after acid wash significantly increased at 2 h when peptide was complexed with AMC6, and at 24 h when complexes are formed with both AMC6 and G4-70/30. The data show mean ± standard deviation of three independent experiments.

Once we determined the peptide-dendrimer association conditions, we assessed whether complexing with the dendrimer or AMC6 facilitated the entry of the peptide into the DCs. Monocyte-derived immature DCs (iDCs) were treated with FITC-labelled peptide, AMC6 nanoparticle or G4-70/30 dendrimer alone or with the complexes AMC6/peptide or G4-70/30 peptide formed at the concentrations used in Figure 2. Following 2 h or 24 h of incubation, the FITC fluorescent signal inside the DCs was measured by flow cytometry and compared to control groups (Figure 3).

DCs treated only with dendrimers showed no fluorescent signal similarly to control untreated cells. Incubation of DCs with the FITC-labelled peptide in absence of nanoparticles, led to the peptide being taken up by almost 20% of the DCs after 2 h of incubation, and by almost 40% of the DCs after 24 h. Complexing with AMC6 nanoparticle efficiently increased peptide uptake after 2 h to almost 70% of the DCs and to 80% after 24 h. On the other hand, G4-70/30 dendrimer did not make a significant difference at the 2 h time-point, but did significantly increase the fluorescent signal in DCs to almost 70% after 24 h, suggesting a slower, but efficient transfection.

Direct proof of the entry of the G4-70/30 dendrimer into the DCs was the intense Cy5.5 fluorescence observed in the cells treated with the labeled G4-70/30 dendrimer (with and without the peptide) in comparison with the untreated cells or cells incubated with the peptide alone or unlabeled G4-70/30 dendrimer (Supplementary Figure S3).

**Figure 4:**
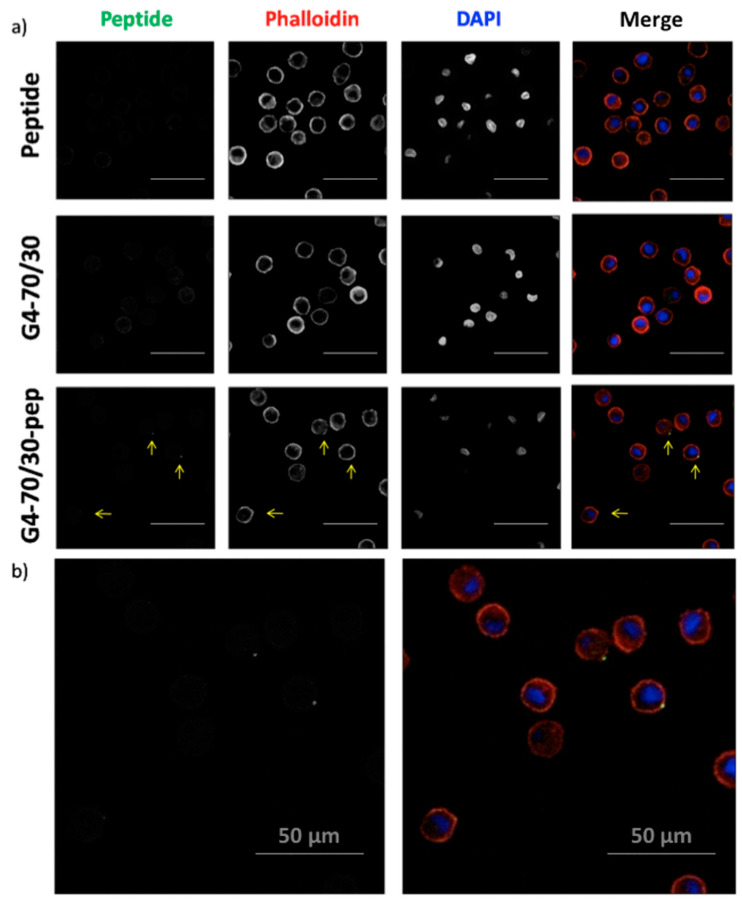
Peptide entry into DCs after 24 h incubation. (**a**) DCs treated with HIV-1 peptide (1 µM), G4-70/30 dendrimer (20 µM) and G4-70/30 (20 µM)-peptide complex, were stained with phalloidin and DAPI and visualized by confocal microscopy. DCs treated with the complex present green fluorescent engrossments where the peptide has entered the cell (arrows). Scale bar: 100 µm (**b**) Enlargement of the images of DCs treated with G4-70/30-peptide complex: green fluorescent peptide (left) and merge (right). Scale bar: 50 µm.

Entry of the complex into the DCs was also directly observed by confocal microscopy. Treated cells from all the sample groups previously described were stained with phalloidin and DAPI to facilitate the identification of the cells and the location of the peptide, after 24 h treatment. No cells were found to have green fluorescent inclusions when treated only with the peptide or in the samples treated with either one of the carriers. In the DCs that were incubated with the complex dendrimer/peptides, we found a significant number of cells containing localized green fluorescent dots close to the cellular plasma membrane, in the intracellular side (Figure 4). Confocal microscopy has less sensitivity for the detection of fluorescence and requires a higher intensity in the samples for it to be detected, which is why we observed no green fluorescence in the cells treated with the peptide alone, and a much lower amounts of labeled cells than expected according to the cytometry data in the sample containing cells treated with the dendrimer-peptide complex. In the case of AMC6-peptide complexes, some cells were also found to have green fluorescent inclusions after 2 h incubation with the complex consistent with the increased in the number of FITC + cells shown in Figure 3a (data not shown). These observations corroborated the results found by flow cytometry, and reinforced the conclusion that both AMC6 nanoparticle and G4-70/30 dendrimer are effective carriers for the p24 peptide into the DCs.

As expected, given the lower entry rate measured by flow cytometry and the lower ability to form complexes, we barely observed green fluorescence in the cells treated with peptides bound to Cy5.5-labeled G4-70/30 dendrimer (Supplementary Figure S4). However, we observed intense fluorescence in the far-red channel (corresponding to Cy5.5), which further demonstrates that the dendrimer enters to almost every DC, meaning that it potentially achieves almost 100% transfection.

### 3.4. Transfection with Complexes Has No Effect on DCs Maturation

**Figure 5:**
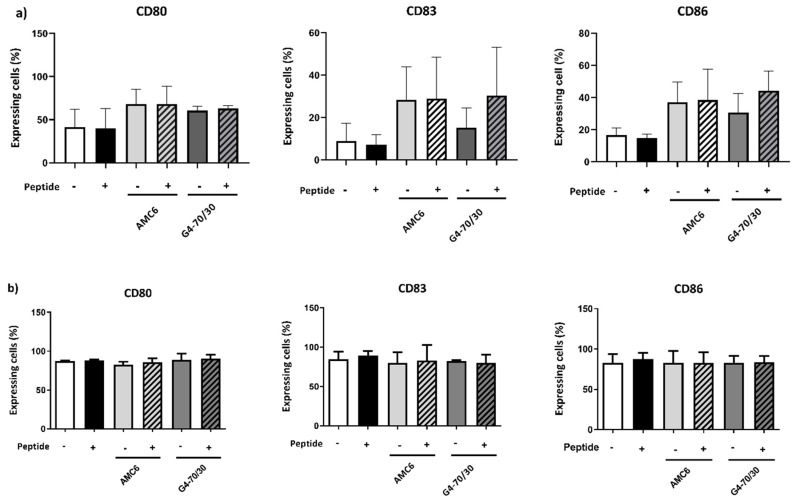
Incubation with the peptide, nanocompounds or complexes does not affect DC maturation. (**a**) iDCs were treated with AMC6 nanoparticle or G4-70/30 dendrimer with and without peptide. (**b**) DCs maturation were induced by treatment with CD40L and TNF-α upon treatment with AMC6 or G4-70/30 with and without peptide. After 48 h, cells were stained with fluorescent antibodies against CD14, CD1a (to detect differentiated DCs) CD80, CD83 and CD86. DC marker expression was measured by flow cytometry. No significant difference was found between untreated and treated cells. Symbol + indicates the presence of the compound and – its absence.

Upon encounter with an antigen, iDCs process and display it bound to MHC for presentation to lymphocytes, and simultaneously undergo maturation. We wondered whether p24 introduced into the DCs by the nanoparticles could act as an antigen and promote the cellular changes required for antigen presentation. With this aim, we analyzed the expression of maturation markers in the DCs 48 h after treatment with AMC6 particle or G4-70/30 dendrimer (both with and without peptide), and compared it to the expression in untreated cells, and cells treated with the peptide alone. We found no significant increase in the expression of CD80, CD83, or CD86 (Figure 5a). However, a tendency to increase the expression was observed in both AMC6-treated cells and G4-70/30 dendrimer, suggesting that the peptide (or the carrier) was being recognized as an antigen and slightly promoting maturation, but not at significant levels.

As DCs need to mature in order to efficiently present antigen to T cells, a therapeutic vaccine would require their maturation. To dodge this problem, we added the known DC maturation factors CD40L (500 ng/mL) and TNF-α (50 ng/mL) to the DCs culture 24 h after treatment. These factors caused maturation in all DCs, independently of the presence of dendrimer or peptide, as suggested by the increased in CD80, CD83, and CD86 expression (Figure 5b).

### 3.5. Treated DCs Induce Some Changes on Autologous PBMCs

**Figure 6:**
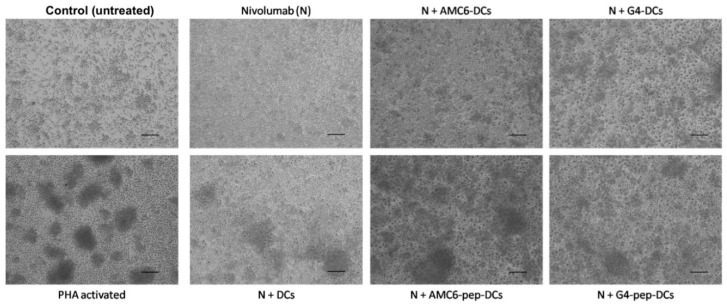
Cell proliferation after MLR observed by brightfield microscopy. PBMCs were cultured for 5 days in the MLR, with DCs treated with AMC6 nanoparticle or G4-70/30 dendrimer, with or without the p42 peptide, in the presence of nivolumab. Untreated DCs were used as control as well as nivolumab alone (without adding DCs). In addition, untreated PBMCs were used as control of no proliferation, and PHA activated PBMCs were used as control for DC activation and proliferation. Scale bar: 100 µm. Abbreviations: G4 = G4-70/30, pep = peptide.

An autologous mixed lymphocyte reaction (MLR) was used to study the effect of the mature DCs transfected with the peptide on lymphocytes. The DCs were mixed at a 1:10 ratio with PBMCs from the same donor, and the effect on the PBMCs was then studied. Analysis of the expression of activation markers in T or B lymphocytes by flow cytometry and measurements of cytokine release showed no effect of the co-culture with DCs on any measured parameter (data not shown). For this reason, we decided to add the monoclonal antibody nivolumab, an anti-human PD1, to the DCs-PBMCs co-culture. Nivolumab potentiates T-cell responses in the presence of a stimulus, i.e., antigen, but not in its absence [28]. After adding nivolumab to the co-culture of PBMCs with autologous DCs treated with peptide-carrier complexes, we observed significant changes in the morphology of the culture under bright-field microscopy indicating cell proliferation (Figure 6). As expected, PBMCs treated with nivolumab alone did not show any proliferation, being the images similar to untreated cells. Similar results were obtained when nivulimab was added to a co-culture of either dendrimer or AMC6-treated DCs with PBMCs. However, when nivulimab was added to a co-culture of DCs treated with the complexes formed by the p42-derived peptide and either the AMC6 nanoparticle or the G4-70/30 dendrimer a marked cell proliferation was observed that was higher in the case of the AMC6/peptide complex suggesting PBMCs activation (Figure 6). This proliferation was similar to that observed for PHA-activated cells used as proliferation control (Figure 6).

### 3.6. Transfected DCs Do Not Modify the Expression of Activation Markers on T or B Cells

**Figure 7:**
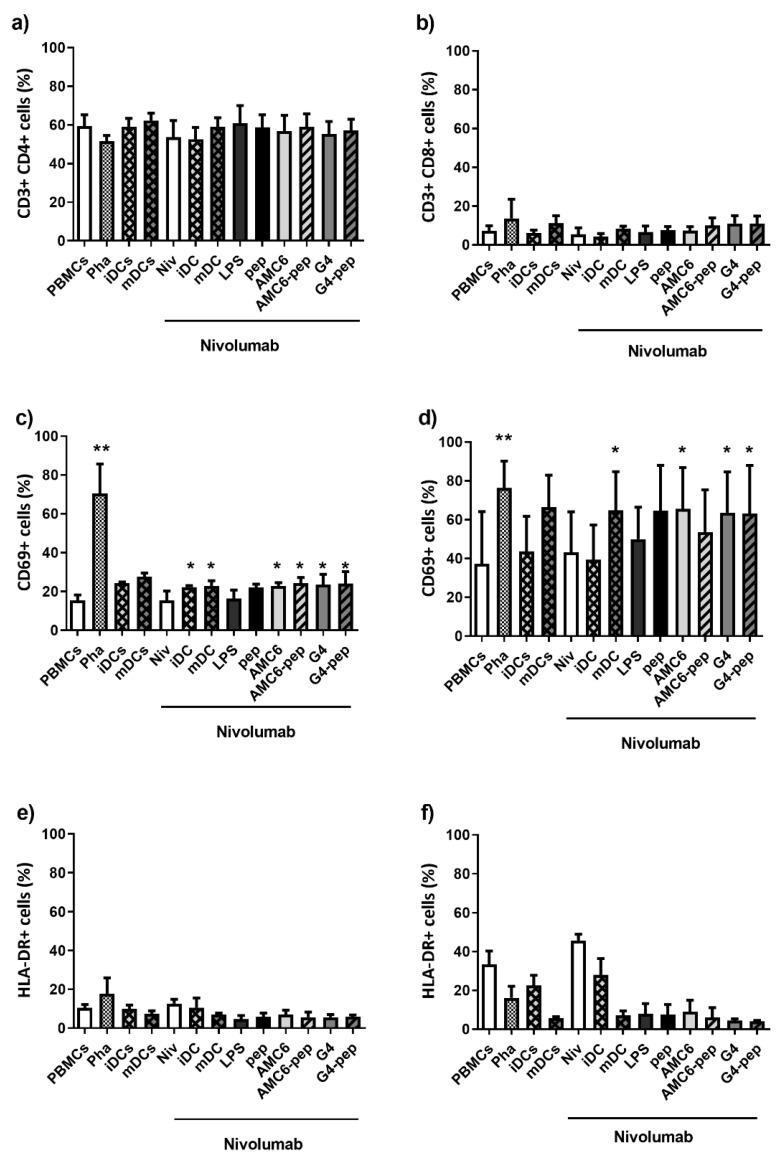
G4-70/30 (20 µM) dendrimer or AMC6 (3 µM) nanoparticle treated DCs cause small phenotypic alteration on CD4^+^ or CD8^+^ T cells after 5 days of co-culture. After co-culture of PBMCs with autologous DCs treated with the nanocompound-peptide complexes, the CD4^+^ and CD8^+^ T cell populations were analyzed by flow cytometry. These graphs show measurements of the population (**a**,**b**), and expression of CD69 early activation marker (**c**,**d**) and HLA-DR (**e**,**f**) on CD4+ (**a**,**c**,**e**) and CD8+ (**b**,**d**,**f**) T cells. Untreated PBMCs and PHA-activated PBMCs (Pha) were negative and positive controls respectively. PBMCs treated with iDCs and mDCs (without nivolumab) were also used as controls. All the other samples were treated with nivolumab. * *p* < 0.05; ** *p* < 0.01 as compared to PBMCs. Abbreviations: Pha = phytohemagglutinin, LPS-DCs = LPS matured DCs, pep = peptide, G4 = G4-70/30.

The observed cell proliferation led us to study the expression of activation markers on T and B cells after MLR. CD3^+^ CD4^+^ T cells showed a slight but statistically significant increase in CD69 (early activation marker) after 3 and 5 days of co-culture with complex-containing DCs (Figure 7a,c). Although this increase is statistically significant and a positive sign of response, it is not considered of biological relevance. These cells showed no significant increase in HLA-DR expression (late activation marker) after 3, 5, or 7 days of co-culture (Figure 7e). Cytotoxic CD3^+^ CD8^+^ T cells showed similar results (Figure 7b,d,f). We also studied cellular levels for CD25, CD71, CD80, CD86, and HLA-DR (well-accepted B cell activation markers) in B lymphocytes and found no significant differences between untreated controls and samples treated with complex-containing DCs (Supplementary Figure S5).

### 3.7. Transfection of DCs with G4-70/30 Dendrimer Increases the Release of TNF-α, IL-2 and IL-12 after Autologous Mixed Lymphocyte Reaction

**Figure 8:**
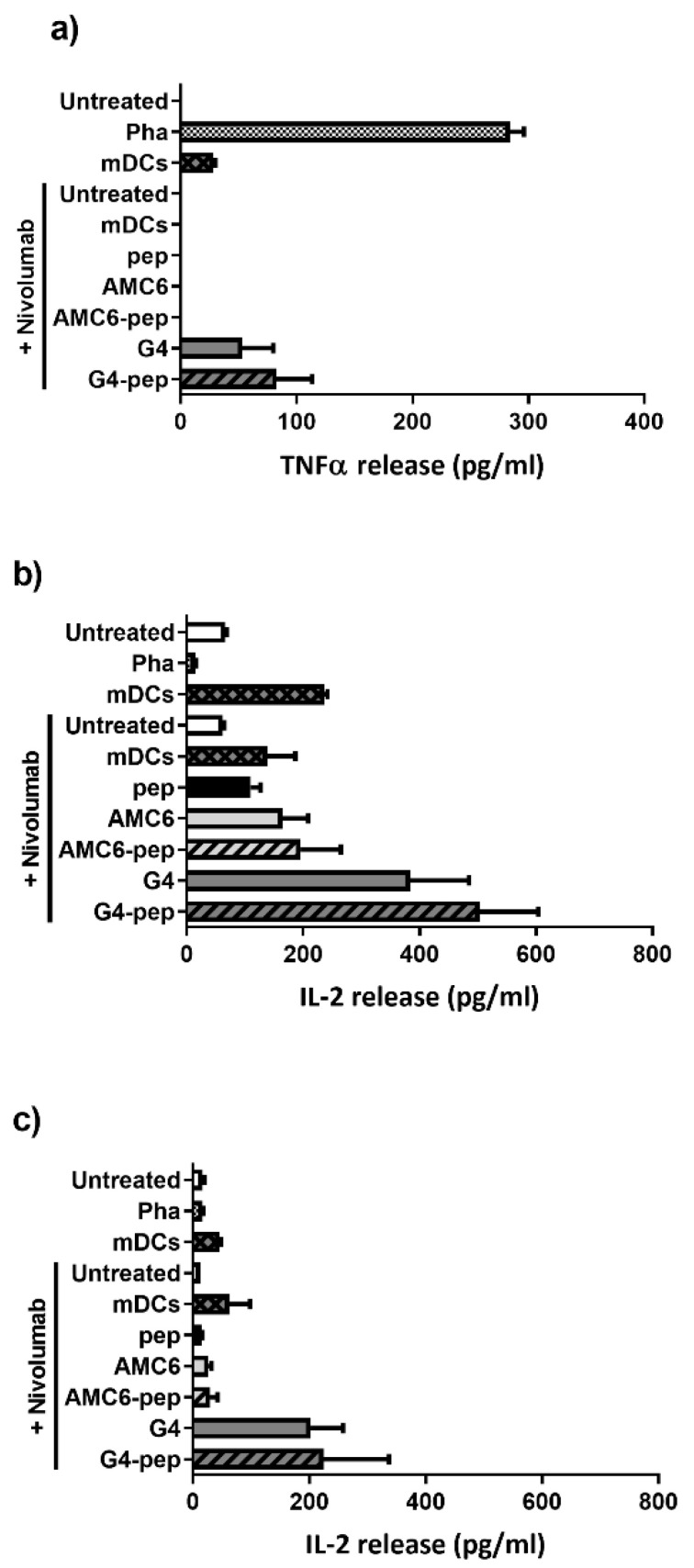
Cytokine release to the MLR media from PBMCs. Cytokine concentration in the extracellular media of the MLR was measured using a DIAplex kit. Significant changes were observed on the expression of IL-12 (**a**), TNF-α (**b**) and IL-2 (**c**). The results shown correspond to the highest concentration of nivolumab used (10 µg/mL) at day 3 (**c**) or day 6 (**a**,**b**) of co-culture. Untreated PBMCs and PHA-activated PBMCs (Pha) are negative and positive controls respectively. PBMCs treated mDCs (without nivolumab) are also used as controls. All the other samples were treated with nivolumab. Abbreviations: Pha = phytohemagglutinin, pep = peptide, G4 = G4-70/30.

Upon activation, CD4^+^ T cells respond to antigen by releasing cytokines, including IL-2 or TNF-α. TNF-α is a cytokine usually released by activated macrophages and CD4+ T cells among other immune cells, which induces apoptosis in infected cells and inhibits viral replication. IL-2 is released by activated CD4+ and CD8+ T cells, NK cells and DCs, and is known to promote T cell differentiation into effector and memory T cells and to stimulate differentiation into Th1 and Th2 lymphocytes while impeding differentiation into Th17 lymphocytes. We measured cytokine release to the media after three and six days of MLR using the DIAplex kit, and compared the cytokine concentration in control groups versus complex-containing-DC-treated samples. We observed a significant increase in TNF-α and IL-2 release in the samples treated with G4-70/30-peptide dendriplexes for three days (Figure 8). Both G4-70/30 dendrimer alone and the peptide/dendrimer complex increased secretion of the proinflammatory cytokines TNF-α and IL-2 suggesting induction of an inflammatory response in the presence of nivolumab (10 µg/mL). On the other hand, at a higher concentration of nivolumab (10 µg/mL), the incubation of PBMCs with DCs containing G4-70/30-peptide complexes led to an increased IL-12 release (Figure 8a). However, we did not find significant differences among any of the groups regarding release of IL-1β, IL-17A, IL-4, IL-10, or IFN-γ cytokines.

## 4. Discussion

A functional cure for HIV-1 infection will require, among other possible therapies, the development of a therapeutic vaccine based on DCs [29,30,31]. The first challenge in the design of a vaccine is the introduction of the viral peptides into DCs, to be recognized as antigens, processed and displayed by a surface MHC complex. Nanoparticles have been shown to be very efficient tools to deliver different compounds such as DNA [32], siRNA [33], and peptides [34] to different cell types. We have taken advantage from this nanoparticle property and use PAMAM G4-70/30 dendrimer and the β-cyclodextrin derivative AMC6 as delivery agents for a HIV-1 p24 protein-derived peptide to mature DCs. The results presented in this study show that both polycationic carriers are efficient for peptide delivery into DCs opening new approaches for potentiating DC antigen presentation.

Both G4-70/30 PAMAM and β-cyclodextrin derivative AMC6 polycationic nanoparticles were able to bind and deliver the antigenic peptide to DCs. We have chosen an antigenic peptide derived from the gag p24 protein since this protein have been shown to be antigenic in several vaccine preparation attempts [14]. We failed in a previous work to induce a robust antigenic response using as delivery agent a cationic phosphorous dendrimer [13]. However, both nanoparticles used in the present work, the PAMAM dendrimer the G4-70/30 PAMAM and the β-cyclodextrin-based nanocompound AMC6 show a much better profile for interacting and delivering peptides to different cell types. This provides the rationale to perform the present work aimed to elicit a strong immune response from human DCs providing a “proof-of-concept” for the use of some nanoparticles as facilitating tools for the function of APCs.

While at 24 h both nanoparticles promote similar peptide uptake by DC, at two hours, the β-cyclodextrin derivative AMC6 nanoparticle was much more efficient. This difference indicates the possible relevance of the uptake pathway for the rate of cargo delivery into the cell [35]. So, it would be reasonable to assume that the presence of mannose in the G4-70/30 PAMAM dendrimer structure would promote its uptake via specific mannose receptors (MMR, DC-SIGN) coupled to clathrin-mediated endocytosis while polycationic amphiphilic cyclodextrins can be taken up by both clathrin-dependent and clathrin-independent mechanisms being the later more relevant in the case of plasmid DNA transfection [35,36]. This difference in the intracellular uptake routes might explain the observed temporal differences in the peptide delivery mediated by the amphiphilic β-cyclodextrin AMC6 or the G4-70/30 PAMAM dendrimer.

The next step for a proper immune response to the delivered peptide consists in stimulating a specific response from lymphocytes after being in contact with the antigen-presenting mature DCs. To achieve DC maturation, we added the known DC maturation factors CD40L (500 ng/mL) and TNF-α (50 ng/mL) to the DC culture 24 h after treatment. These factors caused maturation in all DCs, independently of the presence of G4-70/30 dendrimer or peptide, as indicated by the increase in DCs maturation markers CD80, CD83 and CD86 expression. The autologous MLR, which consists on co-culture of PBMCs with treated DCs differentiated from monocytes from the same patient, serves to test the efficiency of the peptide-containing DCs to present antigen and stimulate a specific immune response. After failing to observe any change in the PBMCs response, we decided to add nivolumab to the MLR culture. Nivolumab (anti-human PD-1) binds PD-1 with high affinity and specificity, and effectively inhibits the interaction with its ligands [37]. PD-1 is a receptor found on T cells, which upon binding to its ligands PD-L1 and PD-L2, inhibits T-cell proliferation and cytokine production. At very low concentrations (~1.5 ng/mL) nivolumab has been shown to enhance T-cell responses in the mixed lymphocyte reaction in vitro in the presence of a stimulus, but not in the absence of stimulus [38], and similar results were found on PBMCs [33]. It is currently approved and used as a treatment for some cancer types [39].

As the bright field images show, this strategy resulted in observable changes in the MLR cultures. The DCs treated with the peptide-carrying nanoparticles induced PBMCs proliferation being AMC6 nanoparticle more effective than the G4-70/30 cationic dendrimer. However, when we analyzed the expression of activation markers on B and T cells by flow cytometry, we only found a statistically, but not biologically significant, increase in the expression of CD69 marker on T cells, and we did not observe significant changes in any of the other markers analyzed. 

Following the lack of expression of activation markers in B and T cells, we decided to study the release of the proinflammatory cytokines TNF-α and IL2 to the extracellular milieu after MLR. TNF-α is a cytokine usually released by activated macrophages and CD4^+^ T cells among other immune cells, which induces apoptosis in infected cells and inhibits viral replication. IL-2 is released by activated CD4^+^ and CD8^+^ T cells, NK cells, and DCs, and is known to promote T cell differentiation into effector and memory T cells and to stimulate differentiation into Th1 and Th2 lymphocytes while impeding differentiation into Th17 lymphocytes. Only the addition to PBMCs of DCs treated with either G4-70/30 dendrimer or the dendriplex dendrimer/HIV peptide induced an increase in the release of the proinflammatory cytokines IL-2 and TNF-α which might suggest a CD4^+^ T cell response. Since no statistical differences were observed between G4-70/30 dendrimer or the dendriplex dendrimer/HIV peptide this might suggest that the G4-70/30 dendrimer might be immunogenic by itself. DCs treated with AMC6 nanoparticle caused no alteration in the cytokine release suggesting lack of proper antigenic presentation mediated by this nanoparticle. However, more studies exploring cytotoxicity mediated by CD8^+^ T cells or antibody release by B cells are needed in order to explain the increase in proliferation and activation observed in the microscopy experiments.

## 5. Conclusions

The work shown here describes the initial steps in the design of a DC-based therapeutic vaccine against HIV-1. Although the preliminary results look promising, further studies including proliferation assays, and measurement of cytotoxicity and antibody release after MLR are needed to evaluate the efficiency and specificity of the vaccine, and to optimize the system to obtain a more desirable response. One way to improve vaccine development would be to deliver to DCs several HIV-1 peptides derived from different proteins (i.e., p24, p160 and Nef), as it may lead to a stronger and more efficient response than trying to drive the immune response against a single antigen. In addition, a better knowledge of the interactions of the polycationic carriers with the molecular mechanisms involved in antigen presentation from uptake into DC to HLA-socket presentation would help to design much more efficient nanocompounds that would facilitate efficient antigen presentation and, accordingly, an efficient immune response.

## Supplementary Materials

The following are available online at https://www.mdpi.com/1999-4923/12/7/656/s1, Figure S1. Chemical structure of G4-70/30 PAMAM dendrimer, Figure S2: Chemical structure of AMC6 nanocompound, Figure S3: Entry of Cy5.5 labeled dendrimer into the DCs observed by flow cytometry, Figure S4: Dendrimer and peptide entry to DCs, Figure S5: G4-70/30 (20 µM) or AMC6 (3 µM) treated DCs cause no phenotypic alteration on B cells after 5 days of co-culture.
