# Supplementary material for: Nanoparticle-Delivered HIV Peptides to Dendritic Cells a Promising Approach to Generate a Therapeutic Vaccine
Source: Pharmaceutics. 2020 Jul 11;12(7):656. doi: 10.3390/pharmaceutics12070656 (PMC7407655; doi:10.3390/pharmaceutics12070656)
Supplement: Supplementary file 1 [file pharmaceutics-12-00656-s001.pdf]

pharmaceutics

Supplementary Materials: Nanoparticle-Delivered HIV Peptides to Dendritic Cells a Promising Approach to Generate a Therapeutic Vaccine

Alba Martín-Moreno, José L. Jímenez Blanco, Jamie Mosher, Douglas R. Swanson, José M. García Fernández, Ajit Sharma, Valentín Ceña and María Angeles Muñoz-Fernández *

Electronic Supplementary Information.

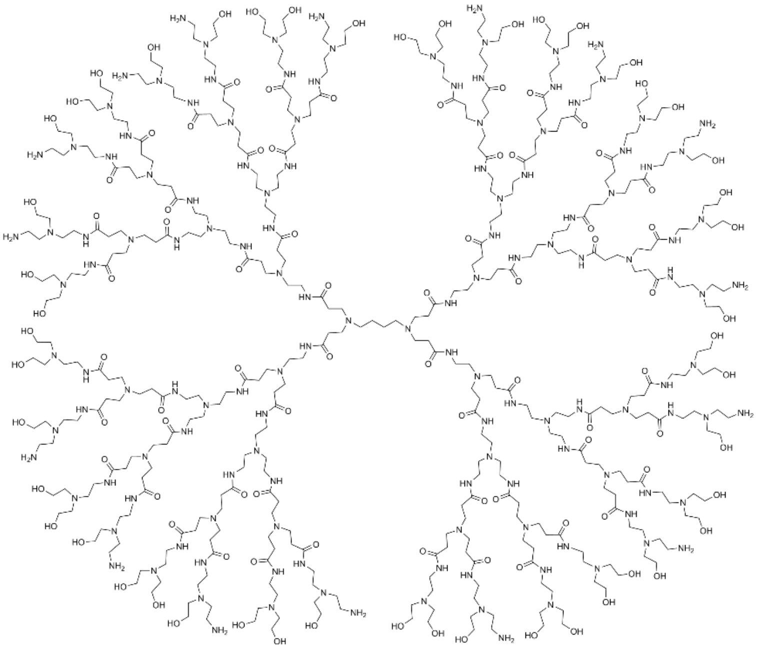

Figure S1. Chemical structure of G4-70/30 PAMAM dendrimer.

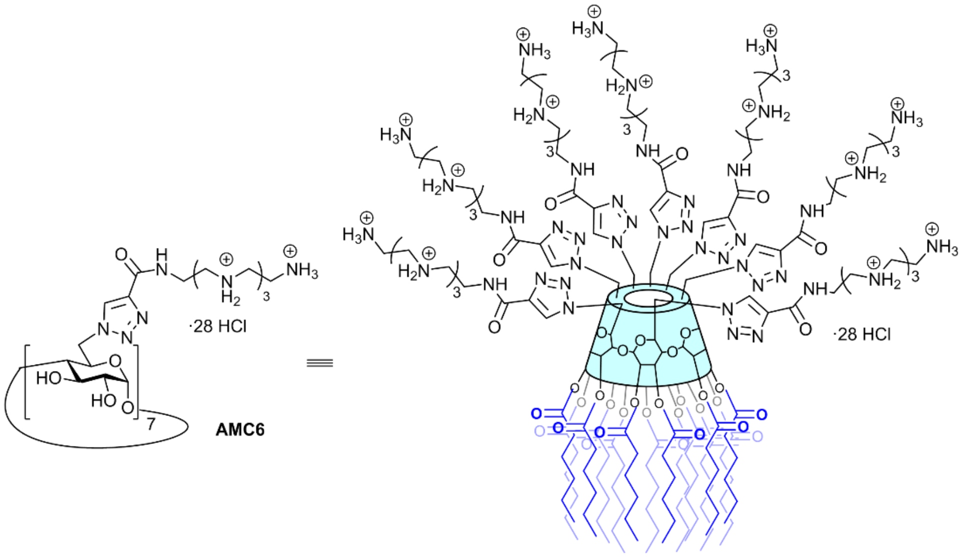

Figure S2. Chemical structure of AMC6 nanocompound.

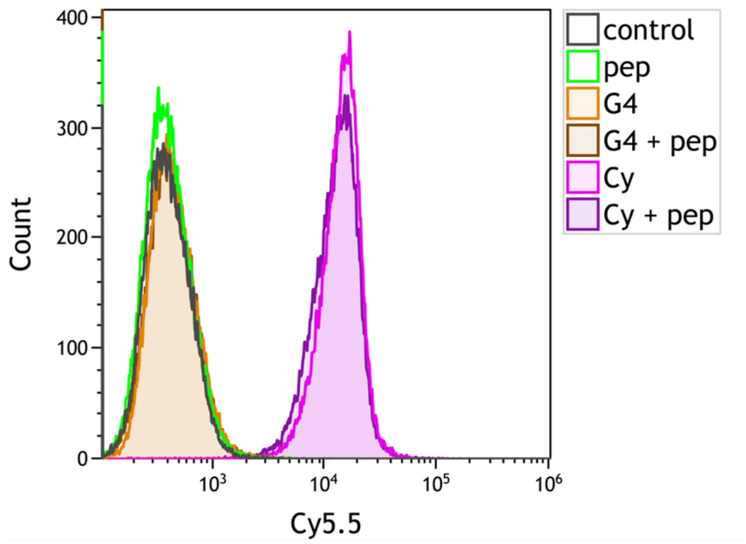

Figure S3. Entry of Cy5.5 labeled dendrimer into the DCs observed by flow cytometry. The dendrimers, peptide and complexes were added to the DCs and, after 2 h, Cy5.5 fluorescence was quantified by flow cytometry. The graph shows the intensity of Cy5.5 fluorescence after acid wash, which significantly increases when the labeled dendrimer is added. Abbreviations: pep = peptide, G4 = G4-70/30, Cy = G4-70/30-Cy5.5.

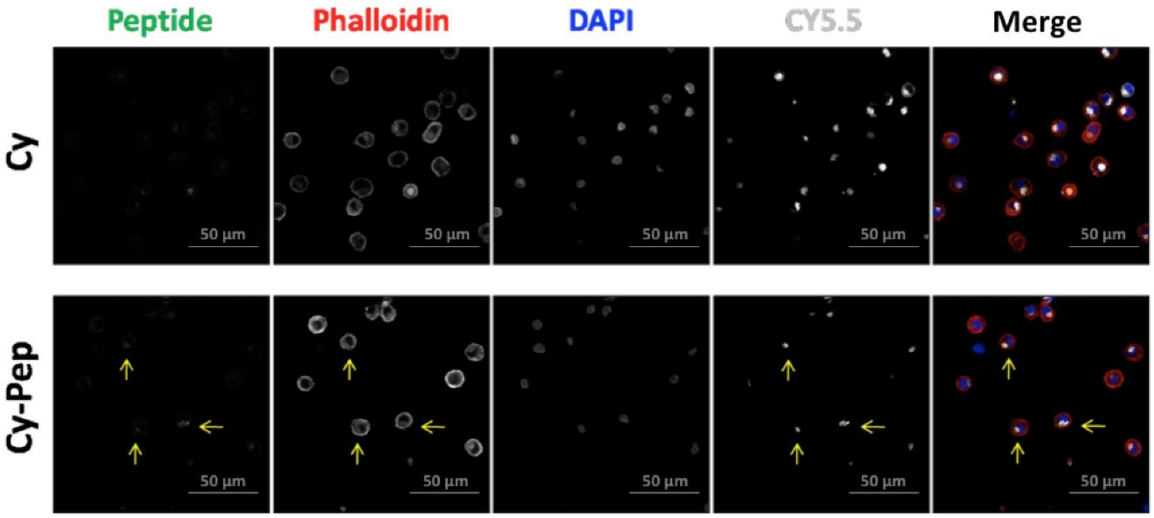

Figure S4. Dendrimer and peptide entry to DCs. DCs treated with G4-70/30-Cy5.5 dendrimer and G4-70/30-Cy5.5-peptide complexes were stained with phalloidin and DAPI and visualized by confocal microscopy. The green fluorescent peptide accumulations co-localize with the Cy5.5 fluorescent dots, suggesting the entry of the whole peptide-dendrimer complex into the DC.

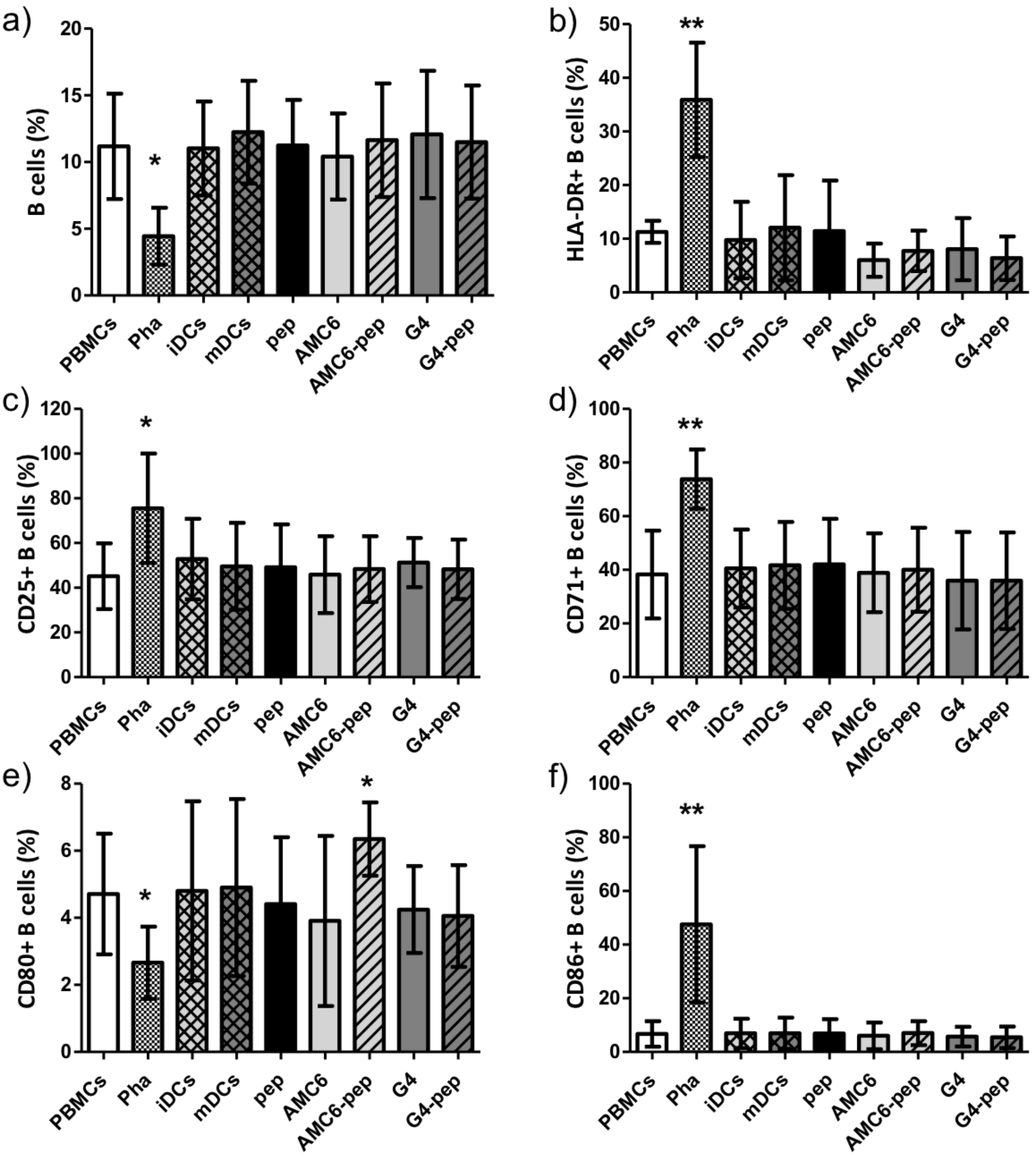

Figure S5. G4-70/30 (20 μM) or AMC6 (3 μM) treated DCs cause no phenotypic alteration on B cells after 5 days of co-culture. After co-culture of PBMCs with autologous DCs treated with the nanocompound-peptide complexes, B cells were analyzed by flow cytometry. These graphs show measurements of the B cell population (**a**), and expression of activation markers: HLA-DR (**b**), CD25 (**c**), CD71 (**d**), CD80 (**e**), and CD86 (**f**). Untreated PBMCs and PHA-activated PBMCs (Pha) are negative and positive controls respectively. PBMCs treated with iDCs and mDCs (without nivolumab) are also used as controls. All the other samples were treated with nivolumab. **p* < 0.05; ***p* < 0.01 as compared to PBMCs. Abbreviations: Pha = phytohemagglutinin, LPS-DCs = LPS matured DCs, pep = peptide, G4 = G4-70/30.
